# Impaired production of immune mediators in dengue virus type 2-infected mononuclear cells of adults with end stage renal disease

**DOI:** 10.1038/s41598-019-56381-3

**Published:** 2019-12-24

**Authors:** Ing-Kit Lee, Zih-Syuan Yang, Hwee-Yeong Ng, Lung-Chih Li, Wen-Chi Huang, Yi-Chun Chen, Ching-Yen Tsai, Chien-Te Lee

**Affiliations:** 1 Division of Infectious Diseases, Department of Internal Medicine, Kaohsiung Chang Gung Memorial Hospital, Kaohsiung, 833 Taiwan; 2 Chang Gung University Medical College, Tao-Yuan, Taiwan; 3 Department of Medical Research, Kaohsiung Chang Gung Memorial Hospital, Kaohsiung, 833 Taiwan; 4 Division of Nephrology, Department of Internal Medicine, Kaohsiung Chang Gung Memorial Hospital, Kaohsiung, 833 Taiwan

**Keywords:** Immunology, Diseases

## Abstract

Chronic kidney disease is an epidemiologically identified risk factor for development of severe dengue in dengue-affected patients. However, available data on the immune pathogenesis in end stage renal disease (ESRD) patients affected by dengue is insufficient. We performed an *in vitro* study to evaluate the sequential immunological reactions and viral load in dengue virus type 2-infected mononuclear cells of patients with ESRD (n = 34) and in healthy controls (n = 30). The concentrations of interleukins (IL)-1 receptor antagonist (Ra), IL-2, IL-6, IL-8, IL-10, IL-12p40, granulocyte-macrophage colony-stimulating factor (GM-CSF), monocyte chemotactic protein-1 (MCP-1), macrophage inflammatory protein-1b (MIP-1b), vascular endothelial growth factor (VEGF), tumor necrosis factor (TNF)-α and viral load cycle threshold (Ct) were measured in the dengue virus type 2-infected mononuclear cells at 6 h, 24 h, 48 h, and 72 h post-infection. We found in the ESRD group significantly higher GM-CSF and IL-2 levels at 6 h post-infection. However, IL-8, IL-10, IL-12p40, TNF-α, MCP-1, and MIP-1b levels were found significantly lower than in the control group. At 24 h, 48 h, and 72 h post-infection, significantly lower levels of IL-1Ra, IL-6, IL-8, IL-10, IL-12p40, TNF-α, MCP-1, and MIP-1b were detected in ESRD group. Concentration of VEGF at 24 h and 48 h, and of GM-CSF at 48 h and 72 h were also found to be lower in ESRD group than in control group. Compared with controls, the viral load Ct values were significantly lower in ESRD group at 6 h and 24 h post-infection No significant difference in viral load Ct values between two groups was found at 48 h and 72 h post-infection. Our study discloses that the expression of immune mediators of dengue-infected mononuclear cells is impaired in ESRD patients.

## Introduction

Dengue is one of the most prevalent arthropod-borne viral diseases worldwide^1^. It is widespread throughout the tropical and subtropical regions of the world^1,2^. An estimate indicates that 390 million cases of dengue occur every year and 3.9 billion people are at a risk of infection^2^. The spectrum of clinical manifestations of dengue ranges from mild nonspecific febrile illness and fever, to the potentially catastrophic complications of severe dengue, as stated in the 2009 World Health Organization guidelines on dengue^1^. The complexity in dengue pathogenesis is an obstacle to the development of anti-viral agents. Thus, the cornerstone of management of dengue is early diagnosis and recognition of severe dengue cases, and timely fluid replacement^1,3^. Severe dengue is characterized by increased capillary permeability leading to extensive plasma leakage, massive bleeding and/or organ involvement that can lead to shock and death^1^. Antibody enhancement^4^, aberrant activation of immune response system along with cytokine production^5^, and host comorbidities^6^ are amongst the postulated pathogenesis for severe dengue. Cytokine cascade plays a major role in the pathogenesis of the grave form of dengue and involves complex interaction between pro- and anti-inflammatory cytokines and chemokines that cause increased vascular permeability, resulting in large fluid losses and subsequent shock^5,7^.

During the 1950’s and 1960’s, dengue was generally considered to be a pediatric disease^8,9^. However, in recent decades, advancing age has been reported to be a risk factor for patients with severe dengue, as the comorbidities associated with ageing pose a substantial risk for mortality in elderly patients^10–12^. Chronic kidney disease has been implicated in the development of severe dengue^13–16^. Thein *et al*., in a study of 108 patients with dengue, reported that renal disorder increased odds of death in adults^17^. Kuo *et al*. reported that patients with chronic kidney disease had a higher mortality rate than those without chronic kidney disease^13^. In a study of 304 adults with dengue, chronic renal disease was found to be a risk factor that contributes to the development of acute kidney injury, which is associated with high morbidity and mortality of the affected patients^18^. Wong *et al*. found that end stage renal disease (ESRD) is a risk factor for acute respiratory failure in dengue affected adults^19^. ESRD is also an important clinical predictor of gastrointestinal bleeding in adult patients with dengue^20^. In fact, patients with ESRD are more susceptible to infections (such as bacterial sepsis) with poor clinical outcomes compared with the general population^21^. In spite of ESRD being an increasingly common disease, there is lack of enough data on the immune pathogenesis in ESRD patients affected by dengue. Thus, thorough investigation is urgently required for the better understanding of immune responses in dengue-affected ESRD patients. In the present study, we aim to explore the sequential immunological reactions and viral load *in vitro* by investigating dengue virus-infected mononuclear cells of adults with ESRD, which would eventually make it possible to target interventions for an improved treatment plan for dengue infection.

## Materials and Methods

### Ethics statement

This study was approved by the Institution Review Board of Kaohsiung Chang Gung Memorial Hospital Medical Center, Taiwan (Document no. 102-5046B). Written informed consent from participants was obtained. This research adhered to the principles of the Declaration of Helsinki.

### Study period and participants

The study was conducted at Kaohsiung Chang Gung Memorial Hospital from January 1, 2017 to December 30, 2017. Volunteers were recruited from those suffering from ESRD, and from healthy controls. Age is controlled in the study. ESRD affected volunteers refer to individuals with chronic kidney disease undergoing hemodialysis therapy thrice a week. A 10 ml sample of blood was obtained from each participant (ESRD and healthy individuals). To avoid the influence of dialyzer on the expression of immune mediators, blood samples from ESRD volunteers were collected before starting the dialysis session. The blood samples of all participants were tested for dengue virus-specific immunoglobulin IgM and IgG antibodies using a dengue blot detection kit (Gene Labs Diagnostics, Singapore), to determine any previous dengue infection^22^. Blood samples were anonymized after completion of demographic data collection.

### Preparation of mononuclear cells

The whole blood from all participants (ESRD and healthy individuals) was separated into plasma and blood cells (i.e., leukocytes and erythrocytes) by centrifugation at 2,500 rpm (150 × g) for 20 minutes. Erythrocytes were removed from blood cells by 4.5% dextran sedimentation. After removal of erythrocytes, leukocytes were further separated into mononuclear cells and neutrophils using a density gradient centrifugation (350 g/30 min in Ficoll-Paque PLUS, Amersham Biosciences Corp.) in accordance with the procedures described elsewhere^23^. After this, mononuclear cells from ESRD and healthy individuals were suspended in RPMI medium (Gibco) and seeded into a 24-well culture plate at a density of 1.0 × 10^6^ cells per well for 1 day at 37 °C.

### Preparation of dengue virus serotype 2 (DENV2)

Three large dengue outbreaks occurred in 2002, 2014 and 2015 in southern Taiwan in which the DENV2 has been the predominant serotype in these outbreaks^24^. In our series, an *in vitro* model with mononuclear cells infected with DENV2 was designed. DENV2 (New Guinea C strain, ATCC) was obtained from the Institute of Preventive Medicine, National Defense Medical Center, Taipei. The virus was propagated in Aedes albopictus C6/36 cells in Eagle’s minimal essential medium (Gibco BRL, Grand Island, N.Y., USA) at 28 °C for 5 days. Virus titers were determined based on a standard plaque forming unit (PFU) assay on Baby hamster kidney-21 cells as described previously^25^. The virus titers were adjusted to 5.0 × 10^6^ PFU/mL in RPMI 1640 (Gibco BRL) medium. To achieve sufficient virus-infected mononuclear cells and avoid excessive cellular apoptosis, we used the multiplicity of infection (MOI) of 5, which has been proven appropriate previously^26^.

### DENV2 infection of mononuclear cells from ESRD and healthy individuals

The mononuclear cells from the 24-well culture plates (at a density of 1.0 × 10^6^ cells per well) were inoculated with DENV2 having MOI of 5, and incubated at 37 °C for 2 hours. The infected mononuclear cells were washed to remove extracellular viruses and cultured in RPMI 1640 medium at 37 °C in a 5% CO2 incubator. The supernatants and cells were harvested and analyzed at various time points after infection (6 h, 24 h, 48 h, and 72 h post-infection) for measurement of responsive immune mediators and viral loads.

### Measurement of immune mediators

In this study, the pro-inflammatory mediators are demonstrated by interleukins (IL)-2, IL-6, IL-12p40, and tumor necrosis factor (TNF)-α.The anti-inflammatory mediators are demonstrated by IL-1 receptor antagonist (Ra), and IL-10. The leukocyte-derived growth and chemotactic factors are demonstrated by granulocyte macrophage colony-stimulating factor (GM-CSF) and IL-8, whereas the monocyte and macrophage chemotactic factors are demonstrated by monocyte chemotactic protein-1 (MCP-1) and macrophage inflammatory protein-1b (MIP-1b). The vascular leakage mediator is demonstrated by vascular endothelial growth factor (VEGF). The concentrations of immune mediators in culture supernatants from the infected mononuclear cells were measured using the FlowMetrix System (Luminex Corporation, Austin, TX, USA), according to the manufacturer’s instructions^27^.

### Determination of DENV2 cycle threshold (Ct) values by real-time reverse transcription-polymerase chain reaction (RT-PCR)

Viral RNA was extracted from cultured mononuclear cells (ESRD and healthy individuals) to measure the Ct value by real-time RT-PCR, as previously described^28^. The forward primer, reverse primer, and TaqMan probe sequences for detecting DENV2 were 5′-GGCTTAGCGCTCACATCCA-3′, 5′-GCTGGCCACCCTCTCTTCTT-3′, and 5′-FAM-CGCCCACCACTATAGCTGCCGGA-TAMRA-3′, respectively.

### Data and statistical analyses

Measurements of immune mediators were expressed as mean value and standard deviation (pg/mL). Viral load was determined by Ct value of RT-PCR. Mann–Whitney U test was used to analyze differences in immune mediators and viral load at various time points (6 h, 24 h, 48 h, and 72 h post-infection) between the ESRD and control groups. A *P* value of <0.05 was considered statistically significant. All analyses were performed using SPSS 13.0 (Chicago, IL, USA).

## Results

### Demographic characteristics of the participants

The cells are obtained from 64 participants, including 34 ESRD patients (14 men and 20 women; median age 59.7 years) and 30 healthy controls (12 men and 18 women; median age, 58.8 years). Among the ESRD affected participants, hypertension (61.7%) was the most common comorbidity, followed by type 2 diabetes mellitus (41.2%). None of the participants in control group have underlying medical condition. All participants tested negative for dengue virus-specific immunoglobulins IgM and IgG antibodies, as determined by dengue blot detection kit^22^.

### Immune profiles in overall ESRD affected participants and controls

**Figure 1:**
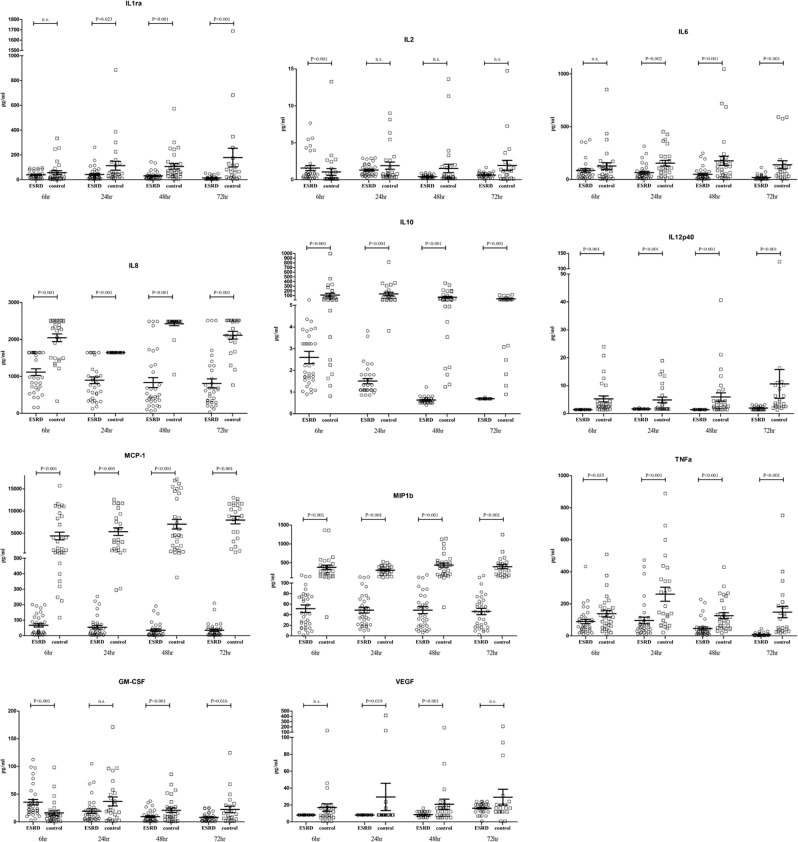
The immune profiles of DENV2-infected mononuclear cells at 6 h, 24 h, 48 h and 72 h post-infection in end stage renal disease (ESRD) group and control group. Data were expressed as mean value and standard deviation (pg/mL).

Figure 1 demonstrates the immune profiles between ESRD group and control group at 6 h, 24 h, 48 h, and 72 h post-infection. Some immune mediators were not detected at a certain time point study. In control group, IL-1Ra, IL-2, IL-6, IL-8, IL-10, IL-12p40, GM-CSF, MCP-1, MIP-1b, VEGF, and TNF-α were not detected in 4 and 7 of the specimens at 24 h and 72 h post-infection, respectively. IL-1Ra, IL-2, IL-6, IL-8, IL-10, IL-12p40, GM-CSF, MCP-1, MIP-1b, VEGF, and TNF-α were not detected in 1 of the specimens in ESRD group at 48 h and 72 post-infection.

At 6 h post-infection, except GM-CSF and IL-2 were significantly higher in the ESRD group than in the control group, the levels of IL-8, IL-10, IL-12p40, TNF-α, MCP-1, and MIP-1b were found to be lower in the ESRD group. At 24 h post-infection, the ESRD group showed significantly lower levels of IL-1Ra, IL-6, IL-8, IL-10, IL-12p40, TNF-α, VEGF, MCP-1, and MIP-1b as compared to the control group. Again at 48 h post-infection, significantly lower levels of IL-1Ra, IL-6, IL-8, IL-10, IL-12p40, GM-CSF, TNF-α, VEGF, MCP-1, and MIP-1b were observed in the ESRD group. At the late phase of infection (72 h post-infection), levels of IL-1Ra, IL-6, IL-8, IL-10, IL-12p40, GM-CSF, TNF-α, MCP-1, and MIP-1b still remained significantly low for ESRD group, as compared to control group.

### Immune profiles in ESRD affected participants without diabetes

**Figure 2:**
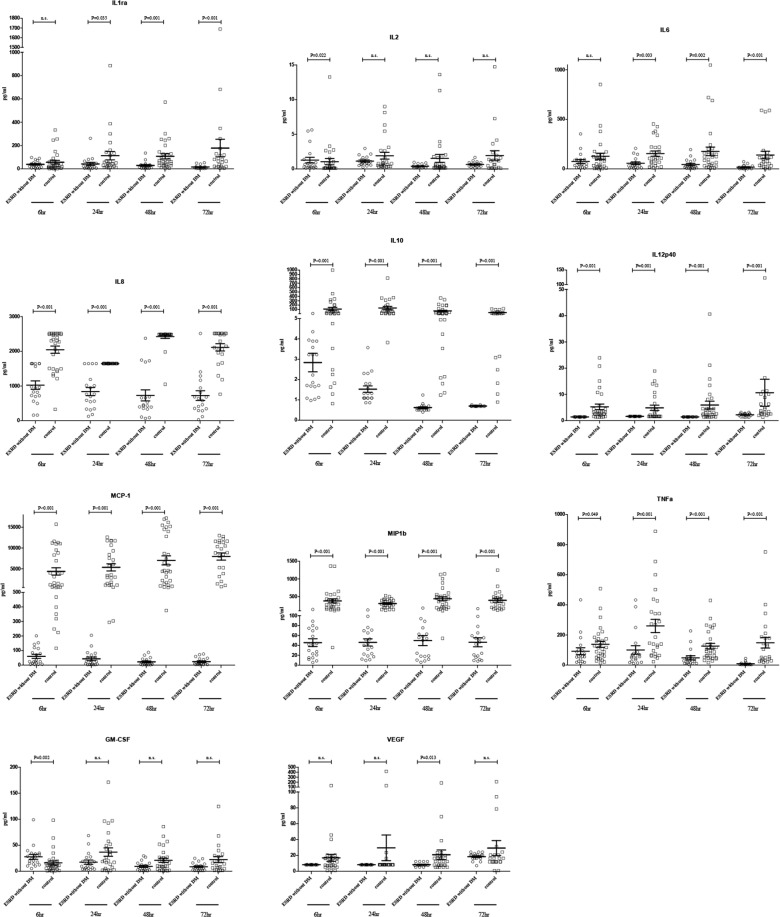
Cytokines/chemokines produced by DENV2-infected mononuclear cells of end stage renal disease (ESRD) without diabetes group and control group at various time points post-infection. Data were expressed as mean value and standard deviation (pg/mL).

Beyond viral factor, the current evidence shown diabetes is a potentially important co-factor that affects the outcome of dengue infection^29^. To eliminate the impact of diabetes in production of immune mediators in ESRD patients, subgroup analysis was performed between ESRD participants without diabetes (n = 19) and control group (n = 30) (Fig. 2). At 6 h post-infection, IL-2 and GM-CSF were detected at higher concentrations, but there was a significant reduction of stimulated mononuclear cell production of IL-8, IL-10, IL-12p40, TNF-α, MCP-1, and MIP-1b in ESRD without diabetes group than in control group. At 24 h, 48 h and 72 h post-infection, the levels of IL-1Ra, IL-6, IL-8, IL-10, IL-12p40, TNF-α, MCP-1, and MIP-1b were still low in ESRD without diabetes group than in the control group. In addition, significantly lower concentration of VEGF was observed at 48 h post-infection in ESRD without diabetes group in comparison to controls.

### DENV2 viral load of ESRD and control groups

**Figure 3:**
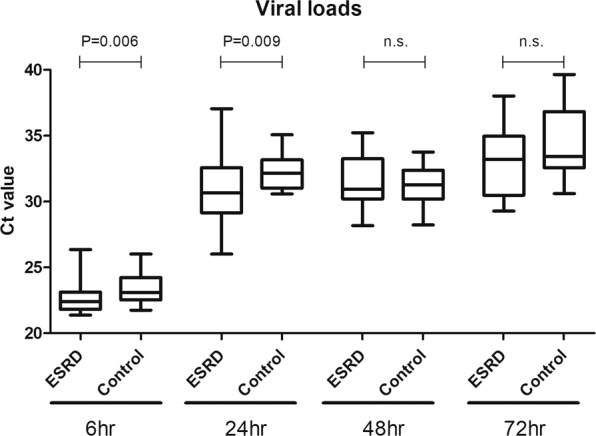
Dengue viral load threshold cycle values in the DENV2-infected mononuclear cells of end stage renal disease (ESRD) group and control group at various time points post-infection.

Viral load Ct values were not detected in 1 of the specimens in ESRD group at 72 h post-infection, and in 1 of the specimens in the control group at 24 h, 48 h and 72 h post-infection, respectively. Compared with controls, the viral load Ct values were significantly lower in ESRD group at 6 h and 24 h post-infection. No significant difference in viral load Ct values between the cultured mononuclear cells from both groups was found at 48 h and 72 h post-infection (Fig. 3).

## Discussion

Chronic kidney disease has been found to be an epidemiologically identified risk factor for development of dengue hemorrhagic fever among dengue patients^13–20^. However, most of the previously published research on dengue immune pathogenesis involved a general population rather than a population with a specific underlying disease such as ESRD^30^. In the present study, eleven immune mediators and viral load Ct values were measured in DENV2-infected mononuclear cells of adults with ESRD, and in healthy participants. Uremic itself presents a complex multidimensional inflammatory condition^31^. Previous studies have shown that T lymphocytes from ESRD patients are dysregulated and the plasma IL-18, IL-6, TNF-α and C-reactive protein concentrations were significantly higher in ESRD patients than those pre-dialysis controls^31^. In our series, apart from high levels of GM-CSF and IL-2 that were observed at 6 h post-infection, impaired production of immune mediators was observed at a later stage post-infection in the ESRD group. This finding is potentially valuable for understanding of the basis of immunopathology reactions and provides new insight in the management of ESRD patients with dengue virus infection.

Patients with dengue virus infection may progress through three clinical phases, namely, the febrile phase, the critical phase and the recovery phase^1^. Our report describes an *in vitro* experiment for dengue virus infected mononuclear cells and analysis of immune mediators at different times after infection, in a setting that mimicked infection in human and the sequential immunological reactions.

The levels of GM-CSF and IL-2 were significantly raised in overall ESRD population as well as those non-diabetic ESRD participants at 6 h post-infection. GM-CSF plays an important role in mediating the innate immune response that stimulates stem cells to produce granulocytes and facilitates development of the immune system to fight against infections^32^. GM-CSF has been reported to increase significantly in patients with severe dengue as compared mild dengue^33^. IL-2 is an important pro-inflammatory cytokine in determining the activation of T-helper 1 response to stimulate cell-mediated immunity^34^. Kurane *et al*. in a study of 69 children showed that the level of IL-2 was highest one day before defervescence in patients with dengue hemorrhagic fever^35^. Although GM-CSF and IL-2 were expressed in higher concentrations at 6 h post-infection in ESRD group, the production of a large number of pro- and anti-inflammatory cytokines as well as chemokines, including IL-8, IL-12p40, TNF-α, MCP-1, MIP-1b, and IL-10 was markedly decreased. These results suggest derangements of immune reaction between pro- and anti-inflammatory cytokines/chemokines in ESRD patients during the early phase of dengue illness.

The hallmark of severe dengue is a transient perturbation in blood vessel integrity, leading to an increase in vascular permeability that results in plasma leakage^1,3^. This change is most likely due to effects of biological mediators as a consequence of complex interactions between dengue virus and host innate and adaptive immune responses^4,5,7^. For example, increased levels of TNF-α and soluble TNF-α receptors as well as VEGF have been reported in dengue hemorrhagic fever cases^36,37^. IL-10, having anti-inflammatory properties counter-regulates the cascade of pro-inflammatory cytokines and it has been reported to correlate with disease severity^38^ Remarkable, the results of the present study showed a significant impairment of IL-1Ra, IL-6, IL-8, IL-10, IL-12p40, TNF-α, MCP-1, and MIP-1b expression at 24 h, 48 h, and 72 h post-infection. VEGF levels at 24 h and 48 h, and GM-CSF levels at 48 h and 72 h were also reported to be low in ESRD group. Impaired cytokines/chemokines expression also has been found in ESRD without diabetes group as well. Our results have been somewhat inconsistent with the reported overwhelming immune activation that is associated with dengue disease severity^5,7^; however, our findings clearly reflect the impairment of immune responses *in vitro* study of DENV2-infected mononuclear cells in ESRD patients. Uremia per se can also alter the immune system in ESRD patients^39^. The complete blood count is not performed in our series. Previous studies, however, have shown that monocytes and monocyte-derived dendritic cells of ESRD patients have impaired endocytosis and maturation^40^. Lisowska *et al*. reported that ESRD patients have lower percentages of CD4+ and CD8+ T cells, as well as B cells in peripheral blood^41^. In fact, several factors influence immunity in ESRD patients, such as uremic toxin, malnutrition, chronic inflammation, and therapeutic dialysis^39–42^. In this regard, our findings and previous reports indicate that ESRD patients have immune dysregulation with impaired expression of immune mediators in case of dengue virus infection.

Although the role of pro- and anti-inflammatory cytokines/chemokines in the pathogenesis of vascular permeability in dengue-affected ESRD patients is uncertain in our series, the impairment of immune responses indicate reduction of the activation of the immune system in dengue-affected ESRD patients that can lead to increased risk of morbidity and mortality in case of secondary bacterial infection. Notably, in a study of 22 severe dengue patients with concurrent bacteremia, 18.2% of them had ESRD^43^. Of 25 adult dengue-affected patients with bacteremia, 16% were found to have ESRD^44^.

Another finding recognized in this study is that significantly lower viral load Ct values at 6 h and 24 h post-infection in ESRD group than controls. However, no significant difference in viral load Ct values between both groups was found at 48 h and 72 h post-infection. The relationship between the dengue viral load and the severity of disease is still controversial^45,46^. Murgue *et al*. had reported that high levels of viral load might contribute to the pathogenesis of dengue hemorrhagic fever^45^. In another study of 128 confirmed adult dengue cases, Chen *et al*. found that viral load was not significantly different between dengue fever and dengue hemorrhagic fever patients^46^. Further studies are needed to determine the role of viral load in the pathogenesis of severe dengue in ESRD patients.

A limitation of the present study is that it is unclear whether similar immunologic responses occur in secondary dengue infection in dengue-affected ESRD patients. Despite the limitation, this is the first study that provides a basis for understanding the role of immune mediators in ESRD population with dengue infection.

In conclusion, the results of our study disclose that impaired immunologic reactions of mononuclear cells to a DENV2 infection in ESRD individuals. This implies that caution needs to be exercised in the immune dysregulation of ESRD population and it might be a key determinant for clinical outcome when treating dengue-affected ESRD patients. Our study highlights that further clinical investigation beyond *in vitro* study is needed to confirm this important finding.

## Data Availability

All data generated or analyzed during this study are included in this published article.

## References

[1] World Health Organization. Dengue: Guidelines for Diagnosis, Treatment, Prevention and Control, New ed. World Health Organization: Geneva, Switzerland (2009). 23762963

[2] Bhatt S (2013). The global distribution and burden of dengue. Nature.

[3] Simmons CP, Farrar JJ, Nguyen VV, Will B (2012). Dengue. N. Engl. J. Med..

[4] Katzelnick LC (2017). Antibody-dependent enhancement of severe dengue disease in humans. Science.

[5] Srikiatkhachorn A, Mathew A, Rothman AL (2017). Immune-mediated cytokine storm and its role in severe dengue. Semin. Immunopathol..

[6] Toledo Joao, George Leyanna, Martinez Eric, Lazaro Adhara, Han Wai Wai, Coelho Giovanini E., Runge Ranzinger Silvia, Horstick Olaf (2016). Relevance of Non-communicable Comorbidities for the Development of the Severe Forms of Dengue: A Systematic Literature Review. PLOS Neglected Tropical Diseases.

[7] Her Z (2017). Severity of plasma leakage is associated with high levels of interferon γ-inducible protein 10, hepatocyte growth factor, matrix metalloproteinase 2 (MMP-2), and MMP-9 during dengue virus infection. J. Infect. Dis..

[8] Cohen SN, Halstead SB (1966). Shock associated with dengue infection. I. Clinical and physiologic manifestations of dengue hemorrhagic fever in Thailand, 1964. J. Pediatr..

[9] Nimmannitya S, Halstead SB, Cohen SN, Margiotta MR (1969). Dengue and chikungunya virus infection in man in Thailand, 1962-1964. I. Observations on hospitalized patients with hemorrhagic fever. Am. J. Trop. Med. Hyg..

[10] Lee IK, Liu JW, Yang KD (2008). Clinical and laboratory characteristics and risk factors for fatality in elderly patients with dengue hemorrhagic fever. Am. J. Trop. Med. Hyg..

[11] Kuo HJ, Lee IK, Liu JW (2018). Analyses of clinical and laboratory characteristics of dengue adults at their hospital presentations based on the World Health Organization clinical-phase framework: Emphasizing risk of severe dengue in the elderly. J. Microbiol. Immunol. Infect..

[12] Hsieh CC (2017). A Cohort Study of Adult Patients with Severe Dengue in Taiwanese Intensive Care Units: The Elderly and APTT Prolongation Matter for Prognosis. PLoS Negl. Trop. Dis..

[13] Kuo MC (2008). Impact of renal failure on the outcome of dengue viral infection. Clin. J. Am. Soc. Nephrol..

[14] Kuo MC (2007). Difficulty in diagnosis and treatment of dengue hemorrhagic fever in patients with chronic renal failure: report of three cases of mortality. Am. J. Trop. Med. Hyg..

[15] Arun Thomas ET, George J, Sruthi D, Vineetha NS, Gracious N (2019). Clinical course of dengue fever and its impact on renal function in renal transplant recipients and patients with chronic kidney disease. Nephrology (Carlton).

[16] Subbiah A (2018). Dengue fever in renal allograft recipients: Clinical course and outcome. Transpl. Infect. Dis..

[17] Thein TL (2013). Risk factors for fatality among confirmed adult dengue inpatients in Singapore: a matched case-control study. PloS one.

[18] Lee IK, Liu JW, Yang KD (2009). Clinical characteristics, risk factors, and outcomes in adults experiencing dengue hemorrhagic fever complicated with acute renal failure. Am. J. Trop. Med. Hyg..

[19] Wang CC (2007). Acute respiratory failure in adult patients with dengue virus infection. Am. J. Trop. Med. Hyg..

[20] Huang WC, Lee IK, Chen YC, Tsai CY, Liu JW (2018). Characteristics and predictors for gastrointestinal hemorrhage among adult patients with dengue virus infection: Emphasizing the impact of existing comorbid disease(s). PLoS One.

[21] Sarnak MJ, Jaber BL (2000). Mortality caused by sepsis in patients with end-stage renal disease compared with the general population. Kidney Int..

[22] Groen J, Koraka P, Velzing J, Copra C, Osterhaus AD (2000). Evaluation of six immunoassays for detection of dengue virus-specific immunoglobulin M and G antibodies. Clin. Diagn. Lab. Immunol..

[23] Chen RF, Yeh WT, Yang MY, Yang KD (2001). A model of the real-time correlation of viral titers with immune reactions in antibody-dependent enhancement of dengue-2 infections. FEMS Immunol. Med. Microbiol..

[24] Chen WJ (2018). Dengue outbreaks and the geographic distribution of dengue vectors in Taiwan: A 20-year epidemiological analysis. Biomed. J..

[25] Chen RF, Wang L, Cheng JT, Yang KD (2012). Induction of IFNα or IL-12 depends on differentiation of THP-1 cells in dengue infections without and with antibody enhancement. BMC Infect. Dis..

[26] Lee IK (2013). Increased production of interleukin-4, interleukin-10, and granulocyte-macrophage colony-stimulating factor by type 2 diabetes’ mononuclear cells infected with dengue virus, but not increased intracellular viral multiplication. Biomed. Res. Int..

[27] Oliver KG, Kettman JR, Fulton RJ (1998). Multiplexed analysis of human cytokines by use of the FlowMetrix system. Clin. Chem..

[28] Memish ZA (2014). Respiratory tract samples, viral load, and genome fraction yield in patients with Middle East respiratory syndrome. J. Infect. Dis..

[29] Lee, I. K., Hsieh, C. J., Lee, C. T. & Liu, J. W. Diabetic patients suffering dengue are at risk for development of dengue shock syndrome/severe dengue: Emphasizing the impacts of co-existing comorbidity(ies) and glycemic control on dengue severity. *J. Microbiol. Immunol. Infect*. pii: S1684-1182(18)30006-9, 10.1016/j.jmii.2017.12.005. [Epub ahead of print] (2018 Jan 31). 10.1016/j.jmii.2017.12.005 30146413

[30] Pang T, Cardosa MJ, Guzman MG (2007). Of cascades and perfect storms: the immunopathogenesis of dengue haemorrhagic fever-dengue shock syndrome (DHF/DSS). Immunol. Cell Biol..

[31] Wong CK (2007). Elevation of pro-inflammatory cytokines, C-reactive protein and cardiac troponin T in chronic renal failure patients on dialysis. Immunol. Invest..

[32] Shi Y (2006). Granulocyte-macrophage colony-stimulating factor (GM-CSF) and T-cell responses: what we do and don’t know. Cell Res..

[33] Patro A. Raj Kumar, Mohanty Sriprasad, Prusty Birendra K., Singh Diwakar K., Gaikwad Sagar, Saswat Tanuja, Chattopadhyay Soma, Das Bidyut K., Tripathy Rina, Ravindran Balachandran (2019). Cytokine Signature Associated with Disease Severity in Dengue. Viruses.

[34] Boyman O, Sprent J (2012). The role of interleukin-2 during homeostasis and activation of the immune system. Nat. Rev. Immunol..

[35] Kurane I (1991). Activation of T lymphocytes in dengue virus infections. High levels of soluble interleukin 2 receptor, soluble CD4, soluble CD8, interleukin 2, and interferon-gamma in sera of children with dengue. J. Clin. Invest..

[36] Mangada MM (2002). Dengue-specific T cell responses in peripheral blood mononuclear cells obtained prior to secondary dengue virus infections in Thai schoolchildren. J. Infect. Dis..

[37] Srikiatkhachorn A (2007). Virus-induced decline in soluble vascular endothelial growth receptor 2 is associated with plasma leakage in dengue hemorrhagic fever. J. Virol..

[38] Boonnak K, Dambach KM, Donofrio GC, Tassaneetrithep B, Marovich MA (2011). Cell type specificity and host genetic polymorphisms influence antibody-dependent enhancement of dengue virus infection. J. Virol..

[39] Girndt M, Sester U, Sester M, Kaul H, Köhler H (1999). Impaired cellular immune function in patients with end-stage renal failure. Nephrol. Dial. Transplant..

[40] Lim WH, Kireta S, Leedham E, Russ GR, Coates PT (2007). Uremia impairs monocyte and monocyte-derived dendritic cell function in hemodialysis patients. Kidney Int..

[41] Lisowska KA (2012). Hemodialysis affects phenotype and proliferation of CD4-positive T lymphocytes. J. Clin. Immunol..

[42] Sterling KA, Eftekhari P, Girndt M, Kimmel PL, Raj DS (2012). The immunoregulatory function of vitamin D: implications in chronic kidney disease. Nat. Rev. Nephrol..

[43] Chen CM (2016). The exploration of risk factors of concurrent bacteraemia in patients critically ill with severe dengue. J. Med. Microbiol..

[44] Chen CM, Chan KS, Chao HC, Lai CC (2016). Diagnostic performance of procalcitonin for bacteremia in patients with severe dengue infection in the intensive care unit. J. Infect..

[45] Wang WK (2003). High levels of plasma dengue viral load during defervescence in patients with dengue hemorrhagic fever: implications for pathogenesis. Virology.

[46] Chen RF (2005). Altered T helper 1 reaction but not increase of virus load in patients with dengue hemorrhagic fever. FEMS Immunol. Med. Microbiol..

